# Kick-starting ovarian cyclicity by using dietary glucogenic precursors in post-partum dairy cows: a review

**DOI:** 10.1080/23144599.2020.1773188

**Published:** 2020-07-09

**Authors:** W. Kaewlamun, B. Grimard, C. Duvaux-Ponter, A. A. Ponter

**Affiliations:** a School of Agricultural Resources, Chulalongkorn University, Bangkok, Thailand; b BREED, Ecole Nationale Vétérinaire d’Alfort, Maisons-Alfort, France; c UVSQ, INRAE, BREED, Université Paris-Saclay, Jouy-en-Josas, France; d INRAE, AgroParisTech, UMR Modélisation Systémique Appliquée Aux Ruminants, Université Paris-Saclay, Paris, France

**Keywords:** Nutrition, reproduction, metabolism, ovarian function, dairy cow

## Abstract

The objective of this review is to describe how dietary glucogenic precursors could stimulate ovarian activity in post-partum dairy cows and improve reproductive success. Although the nutrient requirements for the early resumption of ovarian cycles, and for follicle and embryo development are quantitatively small, reproductive success is deteriorated by post-partum negative energy balance. Since very little glucose is absorbed directly from the digestive tract of ruminants one of the targets for nutritional manipulation could be the glucogenic potential of the diet. This could be achieved by giving rumen-resistant starch or mono-propylene glycol. Both these adaptations increase glucose, insulin and insulin-like growth factor-1 plasma concentrations and stimulate ovarian follicle growth.

## 1. Introduction

The objective of this review was to describe how dietary glucogenic precursors could stimulate ovarian activity in the post-partum (PP) dairy cow and improve reproductive success. As a result of increases in milk production obtained through advances in genetic selection and improved husbandry, reproductive efficiency in dairy cows has declined between 1975–1982 and 1995–1998 from 55.6% to 39.7% [1]. Although this negative trend has recently bottomed out and reproduction has begun to improve due to the inclusion of fertility traits in selection programmes [2], modern dairy cows still require an additional 30d to conceive when comparing results between 1999 and 2010 [3].

Feeding dairy cattle should always be optimized to cover requirements for milk production and maintain good health but it also may be possible through the choice of certain feedstuffs to target a particular physiological function, such as reproduction. The idea of targeting certain aspects of metabolism to stimulate reproduction in dairy cows was first proposed nearly twenty years ago with the use of glucogenic vs. lipogenic diets [4]. Further research has since been conducted.

The first section of this paper will outline the general metabolic context of the dairy cow PP. The second part will describe the rationale behind modifying the diet PP. The final section will describe how glucogenic supply can be increased to improve reproductive success.

## 2. Metabolism in the post-partum dairy cow

In the dairy cow, the negative energy balance (NEB) occurs PP [5] because the increase in feed intake after parturition, is not able to keep up with the rapid rise in energy requirements for milk production [6] even though the cows are fed ad libitum. The requirements for energy and protein of an average European dairy cow at peak milk production are multiplied by 3 to 5 compared to late gestation [7] and the peak in nutrient requirements occurs earlier (at 1 to 2 months PP) compared to the peak in feed intake (at 3 to 4 months PP) therefore inducing NEB [7]. The problem is physiological in relation to a lag in feed intake compared to nutrient requirements.

NEB can also exist in beef cattle PP but the situation is different compared to dairy cows because beef cows are often managed in low input systems. Nutrient requirements are not as high PP but farmers often use low quality forages or limit cow access to good quality forages therefore inducing NEB [7]. The problem is due to the farmer trying to reduce production costs by limiting nutrient intake compared to nutrient requirements.

As a result of NEB, insulin decreases and growth hormone (GH) increases during this period to promote lipolysis [8]. Despite high circulating GH, there is a decrease in insulin-like growth factor-1 (IGF1) because insulin is low and is no longer able to stimulate the expression of the GH receptor 1A. Without this receptor GH cannot stimulate IGF1 production. The somatotropic axis is said to become “uncoupled” [9]. Concomitantly, PP non-esterified fatty acids (NEFA) increase and this can lead to ketosis [10] and hepatic steatosis [11] if NEFA are not completely oxidized or exported. Hepatic steatosis caused by triglyceride accumulation reduces the ability of hepatocytes to synthesize glucose from propionate [12]. In conclusion, PP NEB results in low glucose, insulin and IGF1 and high GH, NEFA, β-hydroxybutyrate (BHB) and liver triglycerides [13].

Homeorhetic modifications occur PP to spare glucose and involve a decrease in insulin concentrations and tissue sensitivity to insulin [14]. Part of the mechanism is raised NEFA which reduce insulin sensitivity by provoking ceramide accumulation in plasma and liver [15,16]. When NEFA are mobilized palmitic acid increases [17] and it is a precursor of ceramides. Plasma ceramides were positively correlated with plasma NEFA and inversely correlated with insulin sensitivity in dairy cows in the peri-partum period [18].

Depending on the tissue, glucose uptake requires insulin (insulin-dependent tissues, adipose tissue, muscle, ovary, hypothalamus) or does not require insulin (non-insulin dependent tissues as brain, heart and udder [19]). Glucose supply is important for ovarian metabolism because insulin-sensitive glucose transporters, GLUT1 and GLUT4, are present in sheep granulosa and theca cells [20] and GLUT4 in cumulus oophorus cells [21] and glucose is taken up by the ovary during the oestrous cycle [22]. Glucose uptake by the ovary may become limited for some cows because insulin is low and insulin-sensitive tissues are less responsive to insulin’s action [23].

In conclusion, the NEB observed after calving activates homeorhetic adjustments to metabolism to divert nutrients towards milk production and this in turn reduces the availability of glucose for reproductive tissues.

## 3. Glucose precursors to improve reproductive efficiency

Changing the composition of the diet or adding mono-propylene glycol (MPG) can increase glucose precursors. The papers published on the effect of a glucogenic supplement (starch or MPG) on reproductive function are summarized in Tables 1–3.

**Table 1.** Summary results of the effect of a dietary glucogenic supplement in the form of starch or mono-propylene glycol (MPG) on reproductive function in grazing post-partum dairy cows

Amount of glucogenic suppl. (ml/cow/d or % diet)		Source of starch	Duration of suppl. (days)	No. Cows /treatment	Metabolic effects				Energy balance	Effect of suppl. on reproductive parameters	
Starch	MPG				Glucose	Insulin	IGF1	NEFA			References
17.8% 38.1%	-	barley-corn	during 36d PP	17	↑	→	↑	→	-	ICO ↓ ICAI → early CR trend ↑	[78, 79]
-	0 200mL 2 x 200mL	-	during 6wk prior to planned mating	580-622	-	-	-	-	-	ICO ↓ CR at 12 and 16wk ↑(200mL>2 x 200mL=0)	[80]
-	0 2 x 250mL	-	during 16wk PP	13-17	→	basal→	→	↓	-	ICO ↓if low BCS at calving final ↑ if BCS low at calving LH pulse frequency ↑	[37]
-	0 2 x 250mL	-	during 21wk PP	13-18	↑	→	-	↓	Good BCS	ICO →	[81]
NSC 22.5% NSC 34.7%	-	control = palm kernel meal-soy meal treatment = barley-corn	during 5-6wk PP stopping 4wk before breeding season	471-478	-	-	-	↓	-	ICO → final CR tended ↓ in one of the herds used in expt → for other herds	[40]

NSC: non-structural carbohydrate, PP: post-partum, ICO: interval calving oestrous, ICAI: interval calving artificial insemination, LH: luteinizing hormone, P4: progesterone concentrations, CR: conception rate, BCS: body condition score, ↑: increase, →: no effect, ↓: decrease, -: not measured.

**Table 2.** Summary results of the effect of a dietary glucogenic supplement in the form of mono-propylene glycol (MPG) on reproductive function in non-grazing post-partum dairy cows

		Amount of glucogenic suppl. (g or mL/cow/d)			Metabolic effects						
Basal diet	Basal diet starch and source	MPG	Duration of suppl. (days)	No. Cows/treatment	Glucose	Insulin	IGF1	NEFA	Energy balance	Effect of suppl. on reproductive parameters	References
TMR maize silage	?	0 mL 250 mL	Between 3–15d PP	9	-	-	-	-	-	ICO → ICAI ↓ CR at 1st AI →	[82]
TMR maize silage	Basal diet maize starch 21%	0 g 225g MPG + 225 g Ca propionate 0 mL 850 mL	During 6 wk PP	10	-	-	-	-	-	ICO ↓ CR at 1st AI ↑ ICF ↓ number AI/fertilization ↓	[83]
TMR	?		−3 to 8d of induced oestrus at 60 DIM	13	-	→	-	→	-	P4 →	[84]
TMR prepartum 66%/33% grass silage or hay/maize silage PP 33%/66% grass silage or lucerne hay/maize silage	Basal diet maize starch 20%	0 mL 300 mL	During 10d prepartum and on days 3, 6, 9 and 12 PP	19–20	-	→	↑	↓	-	At 90d PP number of acyclic cows ↓	[39]
50%/50% maize silage/lucerne hay + concentrates for milk production	Basal diet starch 15%	0 mL 500 mL	Between 7 and 42d PP	16–17	↑	↑	-	↓	No difference between groups	ICO ↓ length of 1st luteal phase ↑ (13.1 vs. 7.3d) P4 secretion → CR 1st AI → CR at 150d PP → Number of AI/conception →	[25]
50%/50% maize silage/ grass silage + concentrates for milk production	Basal diet maize starch 15%	0 mL 500 mL	Between 7 to 35–40d PP	17–18	↑	↑	-	↓	-	No effect on follicle dynamics no effect on LH secretion characteristics no effect on oocytes collected and their quality	[85]
TMR maize silage/grass hay/straw	?	0 mL 6 × 200 mL	Holstein cows at maintenance	17 from start of synchronization and superovulation until after 2^nd^ AI (embryos collection 6d after 2nd AI)	↑	↑	-	-	-	P4 at embryo recovery ↓ ovulation rate ↓ fertilization rate ↓ no effect on recovery rate and quality of recovered embryos	[86]
TMR 50%/50% maize silage/legume hay	Basal diet starch 23.5%	0 mL 500 mL	−10d to +25d PP	28	↑	↑	-	↓	Improved	No effect on follicle dynamics no effect on LH secretion characteristics	[36]
-	-	0 mL 267 mL	super ovulated heifers with AI and embryo collection cross-over	20 treatment over 10d during superovulation and AI period	-	↑	→	-	-	Number of transferable embryos → number of degenerated embryos → number of unfertilized oocytes recovered →	[62]
Hay plus concentrate	Basal diet maize starch 3%	0 mL 400 mL	Super ovulated heifers with OPU and embryo production cross-over	16 treatment over 5d during superovulation	↑	↑	↑	-	No difference in growth rate	Number of follicles, blastocysts and blastocyst quality ↑ in high AMH group	[61]

TMR: total mixed ration, PP: post-partum, ICO: interval calving oestrous, ICAI: interval calving artificial insemination, ICF: interval calving fertilization, DIM: days in milk, P4: progesterone concentrations, CR: conception rate, #: intensive blood sampling after MPG treatment, AMH: anti-Müllerian hormone, ↑: increase, →: no effect, ↓: decrease, -: not measured, ?: basal diet starch % not indicated in publication.

**Table 3.** Summary results of the effect of a dietary glucogenic supplement in the form of starch on reproductive function in non-grazing post-partum dairy cows

Basal diet	Basal diet starch	Amount of glucogenic suppl. (% diet)	Source of starch	Duration of suppl. (days)	No. Cows /treatment	Metabolic effects				Energy balance	Effect of suppl. on reproductive parameters	
		Starch				Glucose	Insulin	IGF1	NEFA			References
50%/50% grass /maize silage concentrates given individually 3.5-12kg	10.4%	26%	maize	between 3wk pre- to 9wk post-calving	42-44 for repro. 25-26for metab.	→	↑ for MultiP cows→ for Prim P cows	-	↓	no difference between groups	ICO ↓ no differences for parameters used to describe reproductive cycles (hormone levels, length …)	[87]
TMR 77%/23% grass/maize silage	8.7%	13.5% 15.9% 18.3% 23.1%	wheat	between 40 and 70d PP	5	→	↑	→	↓	no consistent difference	P4 ↓ 3 to 5d post-ovulation small follicles ↑ pre- and post-ovulation size medium follicle ↓	[45]
TMR 66%/33% grass/maize silage	L = 9.8%	H = 18.2%	wheat	Between calving and 120d PP diets switched at first rise in P4 = HH, HL, LH and LL	15	→	↑	-	-	no difference between groups	total follicles at 60d PP ↑ Number of CL at 60d PP ↓ CR 1st AI ↑ Overall CR ↑	[60]
TMR 66%/33% grass/wheat silage	starch = 19% rumen by-pass starch: 7.1%	starch = 19% rumen by-pass starch: 8.2% 9.4% 10.5% 11.6%	wheat	between 40 and 70d post-calving	6	-	→	→	-	-	no differences for parameters used to describe reproductive cycles (P4 levels, follicle numbers …) before and after synchronisation at 50d PP	[88]
TMR 75%/25% grass/maize silage or 25%/75% grass/maize silage	starch: 11.0% rumen by-pass starch: 4.6%	starch: 18.8% 19.1% 27.1% rumen by-pass starch: 8.0% 8.1% 14.4%	maize or wheat	between 40 and 70dpost-calving	8	-	↑ by starch ↑ by maize vs. grass	→	-	-	no differences for follicle numbers before and after synchronisation at 50d PP P4 ↑ in grass vs. maize silage 3 to 5d post-ovulation	[88]
TMR grass /wheat silage	10%	26%	wheat	between calving and 50d PP	10	-	↑	-	-	-	FSH → follicle developpment → ICO ↓ CR →	[4]
TMR 60%/40% grass/maize silage concentrates for milk production	10.6%	21.5%	maize	between calving and 100d PP 2 diets x 3 dry periods	68-73	→	→	→	→	improved	no difference in normal or abnormal resumption of cycling, ICCL, CL length, cycle length	[89]
TMR 60%/40% grass/maize silage concentrates for milk production	10.6%	21.5%	maize	between calving and 100d PP 2 diets x 3 dry periods	total 130 cows over 6treatments Repetition of Chen et al. [89] with same cows	↑	↑	↑	↓	no difference	no difference in normal or abnormal resumption of cycling and ICAI ICCL shorter Pregnancy rate ↓	[90] [91]
TMR 66%/33% maize silage/lucerne hay	19.20%	35.3%	maize	lactating cows cross-over after 14d	11	-	↑	-	-	improved	Cytochrome P 450 2C and CYP3A activity and mRNA expression ↓ (or tended to ↓) P4 half-life tended to be ↑	[52]

TMR: total mixed ration, PP: post-partum, CL: corpus luteum, ICO: interval calving oestrous, ICAI: interval calving artificial insemination, ICF: interval calving fertilization, P4: progesterone concentrations, CR: conception rate, ICCL: interval calving corpus luteum appearance, ↑: increase, →: no effect, ↓: decrease, -: not measured.

### 3.1. Peri-partum period (ketosis and immunity)

A recent meta-analysis showed that the interval between calving-to-first-service was 8 d longer and calving-to-conception was 16 to 22d longer in cows with subclinical ketosis [24]. In periparturient dairy cows, MPG increased insulin and glucose while decreasing NEFA and BHB [25] and reduced the triacylglycerol content of the liver [26]. Therefore, MPG reduces the risk of cows developing subclinical and clinical ketosis, and hepatic steatosis.

In addition, mastitis and endometritis can become a problem [27] because cows cannot fight oxidative stress and their immune system is depressed PP. Clinical mastitis delays ovarian activity [28], reduces conception rates [29] and increases embryonic losses [30]. In cases of infection, peripheral insulin sensitivity decreases, leading to decreased glucose uptake by insulin-dependent tissues such as skeletal muscle [31], adipose tissue [32] and probably the ovary in order to preserve glucose for the immune system. Indeed, it has been estimated that an activated immune system requires substantial quantities of glucose, 2 kg/day, in addition to lactation requirements [33]. Therefore, the immunologically challenged PP cow may benefit from a dietary supplement of glucose (starch or MPG).

### 3.2. Ovarian activity

#### 3.2.1. Delayed resumption of ovarian cyclicity

Numerous growth factors (insulin and IGF1) and metabolites (glucose) influence gonadotropin-releasing hormone (GnRH) release from hypothalamic neurons [34] and both follicle-stimulating hormone (FSH) and luteinizing hormone (LH) are released from the anterior pituitary in response to GnRH [35]. FSH stimulates follicle recruitment and early follicle growth while pulsatile LH is required for continued growth and the development of the dominant ovulatory follicle. Butler [5] found that NEB is strongly associated with low levels of blood glucose, insulin and IGF1 and at the same time LH pulse frequency is reduced. Glucogenic precursors (starch supplement) did not influence FSH concentrations in non-grazing cows [4] and Butler et al. [36] showed that MPG in non-grazing cows had no effect on LH secretion characteristics. To our knowledge only one publication showed a positive effect of MPG on LH pulse frequency [37] and it was in grazing cows. Therefore, glucogenic precursors do not appear to modify FSH and LH secretion parameters. However, the pulsatile nature of their secretion may make studies difficult to undertake.

Britt [38] suggested that the negative effect of NEB on fertility could be explained by a carry-over effect of some metabolites on follicles during their development from inactive primordial follicles up to ovulation which takes between 60 to 80 days. Exogenous and endogenous lipogenic metabolites are acetate, butyrate and long-chain FA while glucogenic metabolites are propionate and starch. A glucogenic diet given between calving and 50 days PP increased plasma insulin and IGF1 compared with a lipogenic diet and this resulted in a greater proportion of cows ovulating by 50 days PP [4]. Rumen MPG fermentation produces propionate and MPG drenches modify ovarian activity and hormones and metabolites [25]. Indeed, ovarian cycles started earlier in cows given MPG drenches PP compared with controls (38% acyclic vs. 58% acyclic at 90d PP) and IGF1 and cholesterol were higher while NEFA was lower although insulin was unaffected [39]. Butler et al. [36] were however unable to show an effect of MPG drenches on ovarian activity in the calving to 27d PP period. Other groups using grazing cows have also been unable to confirm the positive effect of MPG on reproduction [40].

Insulin stimulates follicle recruitment [41] as well as follicular growth and differentiation [42]. Moreover, insulin stimulates *in vitro* proliferation and function of granulosa [43] and thecal cells [44]. Starch addition to the diet of lactating dairy cows increased insulin concentrations [45] and increased insulin in follicular fluid of preovulatory follicles in high producing dairy cattle [46].

#### 3.2.2. Steroid production

A short luteal phase is often observed during the first oestrus cycle PP. This short luteal phase was prevented by giving MPG drenches which increased insulin and restored normal progesterone (P4) concentrations [25]. In addition, P4 is necessary for the uterine secretion of nutrients and growth factors that are essential for early embryonic development.

Circulating steroid hormone concentrations are affected by their rate of production and clearance (hepatic blood flow and catabolic enzyme activities). In goats, weekly administration of insulin prior to and during gestation increased circulating P4 [47]. Insulin may increase P4 production by stimulating cholesterol uptake across the ovary since there was a strong correlation between glucose and cholesterol uptake by the ovary in ruminants [48].

Moriel et al. [49] showed in ovariectomized cows given a P4 intra-vaginal implant that when dietary treatment increased insulin, P4 concentrations were also higher. P4 is inactivated in the liver by cytochrome P450 2 C (CYP2 C) or cytochrome P450 3A (CYP3A) [50,51]. Elevated insulin concentrations produced by dietary manipulation (high starch vs. high fibre) decreased P4 clearance and prolonged P4 half-life in lactating dairy cows [52] without any changes in liver blood flow. CYP2 C activity was decreased and CYP2 C mRNA expression tended to be decreased, and CYP3A activity tended to be reduced and CYP3A mRNA expression was unaffected (starch vs. fibre [53]). Finally, Lemley et al. [54] demonstrated that MPG or insulin infusion decreased the abundance in liver biopsies of mRNA for enzymes responsible for hepatic P4 catabolism. In conclusion, insulin appears to increase circulating P4 concentrations by increasing cholesterol uptake by the ovary and by reducing hepatic steroid clearance.

### 3.3. Oestrus expression

High producing cows have shorter oestrus periods and lower plasma oestradiol (E2) concentrations than those producing less milk [55]. These observations are partly explained by an increase in hepatic clearance since high milk production is associated with high feed intake [56]. *In vivo* Butler et al. [57] using a hyperinsulinaemic-euglycemic clamp in PP dairy cows showed that NEFA decreased and, IGF1 and E2 increased. Further experiments have also confirmed the positive effect of insulin on E2 production in superovulated goats [58]. Therefore, insulin appears to have positive effects on E2 production and may improve the expression of oestrous.

### 3.4. Pregnancy rate

Although insulin has positive effects on follicle growth, it is important to reduce insulin levels during the insemination period. Indeed, high insulin induced by high starch diets during the insemination period had negative effects on oocyte quality and blastocyst development rate [59] and pregnancy rate tended to be reduced [60]. Gamarra et al. [61] showed that MPG drenches during superovulation in heifers improved the production of grade 1 oocytes, expanded blastocysts and embryos after ovum pick-up (OPU), *in vitro* maturation (IVM), fertilization and culture. The collected oocytes were no longer under the influence of high insulin during fertilization and culture since they had been collected and placed in culture medium. Recently using a similar model, Dupras et al. [62] showed that MPG during superovulation and up to the first 4d after artificial insemination (AI) did not influence the number of transferable embryos collected 6d after AI. This finding supports the conclusions of Fouladi-Nashta et al. [59] and Garnsworthy et al. [60].

In conclusion, increasing glucogenic nutrients in the early PP cow could stimulate follicle growth (via increased glucose, insulin and IGF1), limit lipolysis and ceramide production (via insulin) and support P4 concentrations.

### 3.5. Inconsistencies in results

Not all experiments have shown a positive effect of glucogenic precursors on reproductive success. Several factors are identified to explain theses discrepancies (Tables 1–3): sampling frequency, type of feeding system, the genetic background of the cows and a lot of the studies were under-powered. Infrequent sampling (weekly) often resulted in no visible effect of the glucogenic supplement on circulating hormones and metabolites while frequent sampling did. The feeding system modifies the glucogenic profile of the basal diets. Grazing would provide a more lipogenic profile (high sugar and fibre levels) compared with conserved forages (maize silage). Lastly, the genetic background of the cows was different in the studies: New Zealand Holstein and Jersey-Holstein crosses compared with North American Holstein cows. The latter have been shown to produce more milk and mobilize more body reserves. Part of the effect of an increase in milk production was attributed to a reduction in insulin sensitivity in North American cows compared with New Zealand cows [63].

## 4. Practical suggestions to manipulate insulin concentrations

### 4.1. Limit ketosis and steatosis

It has recently been estimated that the average cost of a case of clinical ketosis and a case of sub-clinical ketosis were respectively, €709 and €150 [64]. Mono-propylene glycol was first reported to be useful in the treatment of ketosis in the 1950’s [65]. McArt et al. [66] showed that oral drenching with MPG decreased hyperketonaemia in early lactation dairy cows. While Rukkwamsuk et al. [67] showed that drenching with 400 mL MPG once daily from 7 days prior to expected calving until 7 days after calving reduced steatosis. Therefore, cows with a higher body condition score (BCS) than recommended prior to calving (≥3.5 on a 5 point scale) could be given (−1 to +2 weeks) MPG daily (300 mL/cow/d [66],) either mixed with the concentrates of the diet or given as a drench. MPG will limit adipose tissue lipolysis and steatosis by stimulating insulin secretion and promoting NEFA catabolism.

### 4.2. Encourage ovarian cyclic activity

The idea is to “kick-start” normal ovarian activity in the period +2 to +8 weeks prior to insemination to improve conception rate [68].

Firstly, increase dietary starch level. High rumen fermentable dietary starch is one of the risk factors for acidosis. Maize and sorghum are high in “rumen protected” starch compared with wheat (195–215 g/kg DM vs. 65 g/kg DM [69]. Sauvant et al. [70] calculated that there was no risk of acidosis if dietary rumen digestible starch was below 25% of dry matter. Climate change, currently characterized by increased atmospheric CO_2_, rising temperatures and above all an alteration in the pattern of precipitation [71], may mean that growing sorghum is easier than maize to provide starch since sorghum is much more resilient to low rainfall than maize [72]. In addition to the choice of grain type, the preparation method [73] as well as maturity of grain at harvest are important [74]. Mature ground or rolled grain is recommended [75]. Rumen resistant starch may not be completely hydrolysed in the small intestine [76] due to starch increasing small intestine viscosity [75]. Therefore, the total starch level of the diet should be lower than 35–40% to avoid acidosis and allow complete starch hydrolysis [77]. To ensure complete hydrolysis the quantity of starch reaching the small intestine should not exceed ≈2.5 kg/d [76].

Secondly MPG could be added to the diet. MPG does not cause acidosis. However, MPG is relatively un-palatable and may reduce feed intake if top-dressed on forage. The time course of MPG action is probably different from starch because its effects on glucose and insulin are relatively short-lived, 2–3 h, and of large amplitude. MPG can either be given as a drench or mixed with a concentrate in an automatic concentrate feeder such as in robotic milking systems. In conclusion, a supplement of ≈300 mL MPG/cow/d [66] can be given to stimulate reproductive function. Nielsen and Ingvartsen [65] concluded in their review that at levels of supplementation below 500 g/d unwanted side-effects should not be observed although individual cow responses were variable.

## 5. Conclusion

Glucogenic treatments have a dual role in the improvement of reproductive success. Firstly, through effects on metabolism and secondly, through direct effects on reproductive function.

Glucogenic treatments affect metabolism by reducing the risk of ketosis and steatosis by decreasing lipomobilisation and stimulating ketone oxidation. Limiting lipomobilisation reduces circulating palmitic acid and ceramide production. The latter can cause insulin-resistance and reduce the availability of glucose for the ovary therefore limiting ovarian function.

Glucogenic precursors appear to affect reproductive function by a local (ovary) rather than central mechanisms since they do not influence FSH and LH secretory characteristics. At the local level glucogenic precursors increase follicle recruitment, growth and differentiation, increase E2 concentrations (through improved granulosa and theca cell proliferation and function) and P4 concentrations (increased secretion by the corpus luteum and reduced clearance by the liver) and generally improve oocyte quality. However, maintaining high insulin around insemination may decrease oocyte quality and embryo survival.

## References

[1] Royal MD, Darwash AO, Flint APF, et al Declining fertility in dairy cattle: changes in traditional and endocrine parameters of fertility. Anim Sci. 2000;70:487–501.

[2] García-Ruiz A, Cole JB, VanRaden PM, et al Changes in genetic selection differentials and generation intervals in US Holstein dairy cattle as a result of genomic selection. Proc Natl Acad Sci. 2016;113:E3995–E4004. 27354521 10.1073/pnas.1519061113 PMC4948329

[3] Berry DP, Wall E, Pryce JE. Genetics and genomics of reproductive performance in dairy and beef cattle. Animal. 2014;8:105–121. 24703258 10.1017/S1751731114000743

[4] Gong G, Lee WJ, Garnsworthy PC, et al Effect of dietary-induced increases in circulating insulin concentrations during the early postpartum period on reproductive function in dairy cows. Reproduction. 2002;123:419–427. 11882019

[5] Butler WR Energy balance relationships with follicular development, ovulation and fertility in postpartum dairy cows. Livestock Prod Sci. 2003;83:211–218.

[6] Grummer RR Impact of changes in organic nutrient metabolism on feeding the transition dairy cow. J Anim Sci. 1995;73:2820–2833. 8582873 10.2527/1995.7392820x

[7] Hoden A, Coulon J-B, Faverdin P. Alimentation des vaches laitières. In: Jarrige J, editor. INRA, Paris. Alimentation des bovins, ovins & caprins. 1988. pp. 135-158.

[8] Lucy MC Regulation of ovarian follicular growth by somatotropin and insulin-like growth factors in cattle. J Dairy Sci. 2000;83:1635–1647. 10908067 10.3168/jds.S0022-0302(00)75032-6

[9] Butler ST, Marr AL, Pelton SH, et al Insulin restores GH responsiveness during lactation-induced negative energy balance in dairy cattle: effects on expression of IGF-I and GH receptor 1A. J Endocrinol. 2003;176:205–217. 12553869 10.1677/joe.0.1760205

[10] Schulz K, Frahm J, Meyer U, et al Effects of prepartal body condition score and peripartal energy supply of dairy cows on postpartal lipolysis, energy balance and ketogenesis: an animal model to investigate subclinical ketosis. J Dairy Res. 2014;81:257–266. 24594287 10.1017/S0022029914000107

[11] Wathes DC, Clempson AM, Pollott GE Associations between lipid metabolism and fertility in the dairy cow. Reprod Fertil Dev. 2013;2013(25):48–61. 10.1071/RD12272 23244828

[12] Overton TR, Drackley JK, Ottemann-Abbamonte CJ, et al Substrate utilization for hepatic gluconeogenesis is altered by increased glucose demand in ruminants. J Anim Sci. 1999;77:1940–1951. 10438042 10.2527/1999.7771940x

[13] Evans ACO, Walsh SW The physiology of multifactorial problems limiting the establishment of pregnancy in dairy cattle. Reprod Fertil Dev. 2012;24:233–237. 10.1071/RD11912 22394963

[14] Bauman DE, Currie WB Partitioning of nutrients during pregnancy and lactation: a review of mechanisms involving homeostasis and homeorhesis. J Dairy Sci. 1980;63:1514–1529. 7000867 10.3168/jds.s0022-0302(80)83111-0

[15] Davis AN, Clegg JL, Perry CA, et al Nutrient restriction increases circulating and hepatic ceramide in dairy cows displaying impaired insulin tolerance. Lipids. 2017;52:771–780. 28836149 10.1007/s11745-017-4287-5

[16] Rico JE, Samii SS, Mathews AT, et al Temporal changes in sphingolipids and systemic insulin sensitivity during the transition from gestation to lactation. PloS One. 2017;12:e0176787. 28486481 10.1371/journal.pone.0176787 PMC5423608

[17] Ponter AA, Douar C, Mialot J-P, et al Effect of underfeeding post-partum Charolais beef cows on composition of plasma non-esterified fatty acids. Anim Sci. 2000;71:243–252.

[18] Rico JE, Bandaru VVR, Dorskind JM, et al Plasma ceramides are elevated in overweight Holstein dairy cows experiencing greater lipolysis and insulin resistance during the transition from late pregnancy to early lactation. J Dairy Sci. 2015;98:7757–7770. 26342987 10.3168/jds.2015-9519 PMC6075710

[19] Vernon RG, Sasaki S Control of responsiveness of tissues to hormones In: Elsevier, editor. Physiological aspects of digestion and metabolism in ruminants. Academic Press; 1991 p. 155–182.

[20] Williams SA, Blache D, Martin GB, et al Effect of nutritional supplementation on quantities of glucose transporters 1 and 4 in sheep granulosa and theca cells. Reproduction. 2001;122:947–956. 11732990

[21] Nishimoto H, Matsutani R, Yamamoto S, et al Gene expression of glucose transporter (GLUT) 1, 3 and 4 in bovine follicle and corpus luteum. J Endocrinol. 2006;188:111–119. 16394180 10.1677/joe.1.06210

[22] Scaramuzzi RJ, Campbell BK, Souza CJH, et al Glucose uptake and lactate production by the autotransplanted ovary of the ewe during the luteal and follicular phases of the oestrous cycle. Theriogenology. 2010;73:1061–1067. 20189236 10.1016/j.theriogenology.2010.01.005

[23] Vernon RG, Faulkner A, Hay WW Jr, et al Insulin resistance of hind-limb tissues *in vivo* in lactating sheep. Biochem J. 1990;270:783–786. 2241910 10.1042/bj2700783 PMC1131801

[24] Raboisson D, Mounié M, Maigné E Diseases, reproductive performance, and changes in milk production associated with subclinical ketosis in dairy cows: A meta-analysis and review. J Dairy Sci. 2014;97:7547–7563. 25306269 10.3168/jds.2014-8237

[25] Miyoshi S, Pate JL, Palmquist DL Effects of propylene glycol drenching on energy balance, plasma glucose, plasma insulin, ovarian function and conception in dairy cows. Anim Reprod Sci. 2001;68:29–43. 11600272 10.1016/s0378-4320(01)00137-3

[26] Studer VA, Grummer RR, Bertics SJ, et al Effect of prepartum propylene glycol administration on periparturient fatty liver in dairy cows. J Dairy Sci. 1993;76:2931–2939. 8227621 10.3168/jds.S0022-0302(93)77633-X

[27] Roche JF The effect of nutritional management of the dairy cow on reproductive efficiency. Anim Reprod Sci. 2006;96:282–296. 16996705 10.1016/j.anireprosci.2006.08.007

[28] Ahmadzadeh A, Frago F, Shafii B, et al Effect of clinical mastitis and other diseases on reproductive performance of Holstein cows. Anim Reprod Sci. 2009;112:273–282. 18554826 10.1016/j.anireprosci.2008.04.024

[29] Chebel RC, Santos JEP, Reynolds JP, et al Factors affecting conception rate after artificial insemination and pregnancy loss in lactating dairy cows. Anim Reprod Sci. 2004;84:239–255. 15302368 10.1016/j.anireprosci.2003.12.012

[30] Santos JE, Cerri RL, Ballou MA, et al Effect of timing of first clinical mastitis occurrence on lactational and reproductive performance of Holstein dairy cows. Anim Reprod Sci. 2004;80:31–45. 15036513 10.1016/S0378-4320(03)00133-7

[31] Lang CH, Dobrescu C, Mészáros K Insulin-mediated glucose uptake by individual tissues during sepsis. Metabolism. 1990;39:1096–1107. 2215256 10.1016/0026-0495(90)90172-9

[32] Song MJ, Kim KH, Yoon JM, et al Activation of Toll-like receptor 4 is associated with insulin resistance in adipocytes. Biochem Biophys Res Commun. 2006;346:739–745. 16781673 10.1016/j.bbrc.2006.05.170

[33] Kvidera SK, Horst EA, Abuajamieh M, et al Glucose requirements of an activated immune system in lactating Holstein cows. J Dairy Sci. 2017;100:2360–2374. 28041733 10.3168/jds.2016-12001

[34] Sinclair KD, Webb R Fertility in the modern dairy heifer In: Garnsworthy PC, editor. Calf and heifer rearing: principles of rearing the modern dairy heifer from calf to calving. Nottingham, UK: Nottingham University Press; 2005 p. 277–306.

[35] Wolfenson D, Inbar G, Roth Z, et al Follicular dynamics and concentrations of steroids and gonadotropins in lactating cows and nulliparous heifers. Theriogenology. 2004;62:1042–1055. 15289046 10.1016/j.theriogenology.2003.12.020

[36] Butler ST, Pelton SH, Butler WR Energy balance, metabolic status, and the first postpartum ovarian follicle wave in cows administered propylene glycol. J Dairy Sci. 2006;89:2938–2951. 16840609 10.3168/jds.S0022-0302(06)72566-8

[37] Chagas LM, Gore PJS, Meier S, et al Effect of monopropylene glycol on luteinizing hormone, metabolites, and postpartum anovulatory intervals in primiparous dairy cows. J Dairy Sci. 2007;90:1168–1175. 17297091 10.3168/jds.S0022-0302(07)71603-X

[38] Britt JH Impacts of early postpartum metabolism on follicular development and fertility. Proc Annu Conv Am Assoc Bovine Pract. 1992;24: 39–43. Auburn, AL.

[39] Formigoni A, Cornil MC, Prandi A, et al Effect of propylene glycol supplementation around parturition on milk yield, reproduction performance and some hormonal and metabolic characteristics in dairy cows. J Dairy Res. 1996;63:11–24. 8655735 10.1017/s0022029900031502

[40] McDougall S, Leane S, Butler ST, et al Effect of altering the type of dietary carbohydrate early postpartum on reproductive performance and milk production in pasture-grazed dairy cows. J Dairy Sci. 2018;101:3433–3446. 29428749 10.3168/jds.2016-12421

[41] Webb R, Campbell BK, Garverick HA, et al Molecular mechanisms regulating follicular recruitment and selection. J Reprod Fert. 1999;supplement 54:33–48. 10692843

[42] Spicer LJ, Echternkamp SE The ovarian insulin and insulin-like growth factor system with an emphasis on domestic animals. Domest Anim Endocrinol. 1995;12:223–245. 7587167 10.1016/0739-7240(95)00021-6

[43] Spicer LJ, Chamberlain CS, Maciel SM Influence of gonadotropins on insulin-and insulin-like growth factor-I (IGF-I) induced steroid production by bovine granulosa cells. Domest Anim Endocrinol. 2002;22:237–254. 12044613 10.1016/s0739-7240(02)00125-x

[44] Spicer LJ, Alpizar E, Echternkamp SE Effects of insulin, insulin-like growth factor I, and gonadotropins on bovine granulosa cell proliferation, progesterone production, estradiol production, and (or) insulin-like growth factor I production in vitro. J Anim Sci. 1993;71:1232–1241. 8505257 10.2527/1993.7151232x

[45] Garnsworthy PC, Lock A, Mann GE, et al Nutrition, metabolism, and fertility in dairy cows: 1. Dietary energy source and ovarian function. J Dairy Sci. 2008;91:3814–3823. 18832203 10.3168/jds.2008-1031

[46] Landau S, Braw-Tal R, Kaim M, et al Preovulatory follicular status and diet affect the insulin and glucose content of follicles in high-yielding dairy cows. Anim Reprod Sci. 2000;64:181–197. 11121895 10.1016/s0378-4320(00)00212-8

[47] Suguna K, Mehrotra S, Agarwal SK, et al Effect of exogenous insulin administration on ovarian function, embryo/fetal development during pregnancy in goats. Anim Reprod Sci. 2009;111:202–213. 18479847 10.1016/j.anireprosci.2008.03.021

[48] Rabiee AR, Lean IJ Uptake of glucose and cholesterol by the ovary of sheep and cattle and the influence of arterial LH concentrations. Anim Reprod Sci. 2000;64:199–209. 11121896 10.1016/s0378-4320(00)00208-6

[49] Moriel P, Scatena TS, Sá Filho OG, et al Concentrations of progesterone and insulin in serum of nonlactating dairy cows in response to carbohydrate source and processing. J Dairy Sci. 2008;91:4616–4621. 19038937 10.3168/jds.2008-1286

[50] Murray M Microsomal cytochrome P450-dependent steroid metabolism in male sheep liver. Quantitative importance of 6b-hydroxylation and evidence for the involvement of a P450 from the IIIA subfamily in the pathway. J Steroid Biochem Mol Biol. 1991;38:611–619. 2039754 10.1016/0960-0760(91)90320-5

[51] Murray M Participation of a cytochrome P450 enzyme from the 2C subfamily in progesterone 21-hydroxylation in sheep liver. J Steroid Biochem Mol Biol. 1992;43:591–593. 1419895 10.1016/0960-0760(92)90248-h

[52] Lemley CO, Wilmoth TA, Tager LR, et al Effect of a high cornstarch diet on hepatic cytochrome P450 2C and 3A activity and progesterone half-life in dairy cows. J Dairy Sci. 2010b;93:1012–1021. 20172221 10.3168/jds.2009-2539

[53] Lemley CO, Vonnahme KA, Tager LR, et al Diet-induced alterations in hepatic progesterone (P4) catabolic enzyme activity and P4 clearance rate in lactating dairy cows. J Endocrinol. 2010a;205:233–241. 20223860 10.1677/JOE-10-0042

[54] Lemley CO, Butler ST, Butler WR, et al Insulin alters hepatic progesterone catabolic enzymes cytochrome P450 2C and 3A in dairy cows. J Dairy Sci. 2008;91:641–645. 18218751 10.3168/jds.2007-0636

[55] Lopez H, Satter LD, Wiltbank MC Relationship between level of milk production and estrous behavior of lactating dairy cows. Anim Reprod Sci. 2004;81:209–223. 14998648 10.1016/j.anireprosci.2003.10.009

[56] Sangsritavong S, Combs DK, Sartori R, et al High feed intake increases liver blood flow and metabolism of progesterone and estradiol-17beta in dairy cattle. J Dairy Sci. 2002;85:2831–2842. 12487450 10.3168/jds.S0022-0302(02)74370-1

[57] Butler ST, Pelton SH, Butler WR Insulin increases 17ß-estradiol production by the dominant follicle of the first postpartum follicle wave in dairy cows. Reproduction. 2004;127:537–545. 15129009 10.1530/rep.1.00079

[58] Selvaraju S, Agarwal SK, Karche SD, et al Ovarian response, embryo production and hormonal profile in superovulated goats treated with insulin. Theriogenology. 2003;59:1459–1468. 12527092 10.1016/s0093-691x(02)01196-2

[59] Fouladi-Nashta AA, Gutierrez CG, Garnsworthy PC, et al Effects of dietary carbohydrate source on oocyte/embryo quality and development in high-yielding, lactating dairy cattle. Biol Reprod. 2005;Special issue:135–136.

[60] Garnsworthy PC, Fouladi-Nashta AA, Mann GE, et al Effect of dietary-induced changes in plasma insulin concentrations during the early postpartum period on pregnancy rate in dairy cows. Reproduction. 2009a;137:759–768. 19129370 10.1530/REP-08-0488

[61] Gamarra G, Ponsart C, Lacaze S, et al Dietary propylene glycol and in vitro embryo production after ovum pick-up in heifers with different anti-Müllerian hormone profiles. Reprod Fertil Dev. 2015;27:1249–1261. 25226865 10.1071/RD14091

[62] Dupras R, Mills L, Robert G, et al Can propylene glycol modulate insulin and insulin-like growth factor-1 in superovulated dairy heifers? Reprod Fert Dev. 2018;31:171.

[63] Chagas LM, Lucy MC, Back PJ, et al Insulin resistance in divergent strains of Holstein-Friesian dairy cows offered fresh pasture and increasing amounts of concentrate in early lactation. J Dairy Sci. 2009;92:216–222. 19109281 10.3168/jds.2008-1329

[64] Steeneveld W, Amuta P, van Soest FJ, et al Estimating the combined costs of clinical and subclinical ketosis in dairy cows. Plos One. 2020;15:e0230448. 32255789 10.1371/journal.pone.0230448 PMC7138322

[65] Nielsen NI, Ingvartsen KL Propylene glycol for dairy cows. A review of the metabolism of propylene glycol and its effects on physiological parameters, feed intake, milk production and risk of ketosis. Anim Feed Sci Technol. 2004;115:191–213.

[66] McArt JAA, Nydam DV, Ospina PA, et al A field trial on the effect of propylene glycol on milk yield and resolution of ketosis in fresh cows diagnosed with subclinical ketosis. J Dairy Sci. 2011;94:6011–6020. 22118089 10.3168/jds.2011-4463

[67] Rukkwamsuk T, Rungruang S, Choothesa A, et al Effect of propylene glycol on fatty liver development and hepatic fructose 1, 6 bisphosphatase activity in periparturient dairy cows. Livestock Prod Sci. 2005;95:95–102.

[68] Butler WR, Smith RD Interrelationships between energy balance and postpartum reproductive functions in dairy cattle. J Dairy Sci. 1989;72:767–783. 2654227 10.3168/jds.S0022-0302(89)79169-4

[69] Sauvant D, Chapoutot P, Archimède H La digestion des amidons par les ruminants et ses conséquences. INRA Product Anim. 1997;7:115–124.

[70] Sauvant D, Giger-Reverdin S, Peyraud JL Digestive welfare and rumen acidosis In: Sauvant D, Delaby L, Nozière, editors. INRA feeding system for ruminants, pp. 213-217. Wageningen, The Netherlands: Wageningen Academic Publishers; 2017.

[71] Getachew G, Putnam DH, De Ben CM, et al Potential of sorghum as an alternative to corn forage. Am J Plant Sci. 2016;7:1106–1121.

[72] Schlegel AJ, Assefa Y, O’Brien D, et al Comparison of corn, grain sorghum, soybean, and sunflower under limited irrigation. Agron J. 2016;108:670–679.

[73] Moharrery A, Larsen M, Weisbjerg MR Starch digestion in the rumen, small intestine, and hind gut of dairy cows - A meta-analysis. Anim Feed Sci Technol. 2014;192:1–14.

[74] Peyrat J, Baumont R, le Morvan A, et al Effect of maturity and hybrid on ruminal and intestinal digestion of corn silage in dairy cows. J Dairy Sci. 2016;99:258–268. 26585483 10.3168/jds.2015-9466

[75] Mir PS, McAllister TA, Gibb DJ, et al Dietary oil rich in polyunsaturated fatty acids for ruminants: post-ruminal digesta characteristics and their implications on production. Can J Anim Sci. 2006;86:159–170.

[76] Reynolds CK, Humphries DJ, Van Vuuren AM, et al Considerations for feeding starch to high-yielding dairy cows. Nottingham: Proceedings of the 46th University of Nottingham Feed Conference; 2014 p. 9–11.

[77] Hoover WH, Miller TK Optimising carbohydrate fermentation in the rumen Proceedings of the Sixth Annual Florida Ruminant Nutrition Symposium Gainesville, Florida: University of Florida; 1995 p. 89–95.

[78] Burke CR, Kay JK, Phyn CVC, et al Effects of dietary nonstructural carbohydrates pre-and postpartum on reproduction of grazing dairy cows. J Dairy Sci. 2010;93:4292–4296. 20723702 10.3168/jds.2009-2869

[79] Roche JR, Kay JK, Phyn CVC, et al Dietary structural to nonfiber carbohydrate concentration during the transition period in grazing dairy cows. J Dairy Sci. 2010;93:3671–3683. 20655437 10.3168/jds.2009-2868

[80] Chagas LM, Tunon GE, Taufa VK, et al Reproductive performance of pasture-fed dairy cows supplemented with monopropylene glycol. N Z Vet J. 2010;58:17–22. 20200571 10.1080/00480169.2010.65056

[81] Chagas LM, Gore PJS, Graham G, et al Effect of restricted feeding and monopropylene glycol postpartum on metabolic hormones and postpartum anestrus in grazing dairy heifers. J Dairy Sci. 2008;91:1822–1833. 18420613 10.3168/jds.2007-0339

[82] Yildiz A, Erisir Z Effect of propylene glycol on fertility of postpartum dairy cows experiencing seasonal heat stress. Indian J Anim Res. 2016;50:27–30.

[83] Matras J, Klebaniuk R, Kowalczuk-Vasilev E Impact of glucogenic additive in transition dairy cow diets of varying ruminal starch degradability on yield and composition of milk and reproductive parameters. Czech J Anim Sci. 2012;57:301–311.

[84] Mesilati-Stahy R, Malka H, Argov-Argaman N Influence of glucogenic dietary supplementation and reproductive state of dairy cows on the composition of lipids in milk. Animal. 2015;9:1008–1015. 25691235 10.1017/S1751731115000099

[85] Rizos D, Kenny DA, Griffin W, et al The effect of feeding propylene glycol to dairy cows during the early postpartum period on follicular dynamics and on metabolic parameters related to fertility. Theriogenology. 2008;69:688–699. 18262261 10.1016/j.theriogenology.2007.12.001

[86] Hackbart KS, Bender RW, Carvalho PD, et al Effects of propylene glycol or elevated luteinizing hormone during follicle development on ovulation, fertilization, and early embryo development. Biol Reprod. 2017;97:550–563. 28575154 10.1093/biolre/iox050 PMC6248555

[87] van Knegsel ATM, Van den Brand H, Dijkstra J, et al Effect of glucogenic vs. lipogenic diets on energy balance, blood metabolites, and reproduction in primiparous and multiparous dairy cows in early lactation. J Dairy Sci. 2007;90:3397–3409. 17582125 10.3168/jds.2006-837

[88] Garnsworthy PC, Gong JG, Armstrong DG, et al Effect of site of starch digestion on metabolic hormones and ovarian function in dairy cows. Livest Sci. 2009b;125:161–168.

[89] Chen J, Soede NM, van Dorland HA, et al Relationship between metabolism and ovarian activity in dairy cows with different dry period lengths. Theriogenology. 2015;84:1387–1396. 26282444 10.1016/j.theriogenology.2015.07.025

[90] Chen J, Remmelink GJ, Gross JJ, et al Effects of dry period length and dietary energy source on milk yield, energy balance, and metabolic status of dairy cows over 2 consecutive years: effects in the second year. J Dairy Sci. 2016;99:4826–4838. 26995119 10.3168/jds.2015-10742

[91] Chen J, Soede NM, Remmelink GJ, et al Relationships between uterine health and metabolism in dairy cows with different dry period lengths. Theriogenology. 2017;101:8–14. 28708519 10.1016/j.theriogenology.2017.06.017

